# Anesthetic management of spontaneous cervical epidural hematoma during pregnancy: a case report

**DOI:** 10.1186/s13256-017-1335-y

**Published:** 2017-06-26

**Authors:** Mehdi Samali, Abdelghafour Elkoundi, Achraf Tahri, Mustapha Bensghir, Charki Haimeur

**Affiliations:** Department of Anesthesiology and Intensive Care, Military Hospital Mohammed 5 Rabat, Faculty of Medicine and Pharmacy of Rabat, University Mohammed 5, Rabat, Morocco

**Keywords:** Spontaneous cervical epidural hematoma, Pregnancy, Anesthesia, Fiberoptic bronchoscope intubation

## Abstract

**Background:**

Spontaneous spinal epidural hematoma during pregnancy is a quite rare event requiring emergent decompressive surgery in the majority of cases to prevent permanent neurological damage. Therefore, there is little data in the literature regarding anesthetic management of cervical localization during pregnancy. The potential for difficult airway management with the patient under general anesthesia is one of the major concerns that needs to be addressed to prevent further cord compression. Anesthetic management should also include measures to maintain the mean arterial pressure to improve spinal cord perfusion. Furthermore, spine surgery in pregnant patients needs special consideration in terms of positioning and in the postoperative period.

**Case presentation:**

We present a case of a 35-year-old white woman at 21 weeks of gestation with a spontaneous cervical epidural hematoma. Fiberoptic bronchoscope-guided nasal intubation was a safe option to ensure a higher rate of successful endotracheal intubation while minimizing the risk of aggravating the injury. Her care posed other multiples challenges that required a multidisciplinary team approach.

**Conclusions:**

The case of our patient serves as a reminder of this rare condition and its implications regarding anesthesia.

## Background

Spontaneous cervical epidural hematoma (SCEH) during pregnancy is a rare condition; only ten cases have been reported in the literature to date. The aim of this case report was to present an anesthetic approach in surgery performed in a pregnant woman at 21 weeks of gestation with an SCEH.

## Case presentation

A 35-year-old white woman (weight 75 kg, height 160 cm), gravida 3 para 2, presented to our hospital at 21 weeks of gestation with paraplegia. She had no significant medical or anesthetic history; in particular, she had no history of trauma, and she had not been taking aspirin or anticoagulants. She had been receiving regular care from an obstetrician during pregnancy, which had progressed normally. Twenty-four hours before her admission, the patient had developed a sudden onset of severe interscapular pain radiating to her neck with sudden weakness and paresthesia in both the upper and lower limbs. The patient was confined to bed, and she did not experience any improvement, so she decided to consult in our hospital.

Her physical examination revealed flaccid paralysis in both legs. Her lower limb power bilaterally was grade 0/5. In her right and left higher limbs, power was grades 2/5 and 3/5, respectively. Her upper level with sensitivity was C7–T1. All her deep tendon reflexes were absent. Her plantar response was bilaterally mute. She also had urinary sphincter disturbances. The rest of her physical examination was unremarkable.

**Fig. 1:**
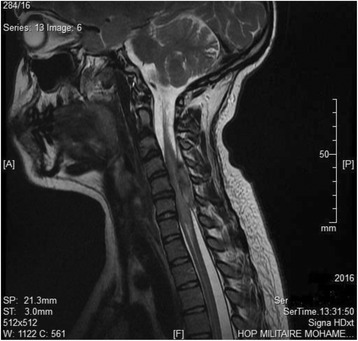
T2-weighted sagittal magnetic resonance image of the cervical spine showing an epidural hematoma located dorsal to the cord extending from C3 to C6 levels, causing obliteration of the spinal subarachnoid space and pushing the cord anteriorly against the vertebral bodies

The results of hematological investigations were within normal ranges: serum potassium 4 mEq/L, hemoglobin 14 g/dl, platelets 180,000/mm^3^, international normalized ratio 1.2, and prothrombin time 11 seconds. Obstetrical ultrasound revealed a normal pregnancy at 21 weeks of gestation and a fetal heart rate (HR) of 140 beats per minute. Magnetic resonance imaging of the cervical and dorsal spine revealed a cervical spine epidural hematoma extending from levels C3 to C6 with severe spinal cord compression (Fig. 1).

An anesthesiologist, a neurosurgeon, and an obstetrician urgently reviewed the case. After discussion with the patient, it was decided to perform spinal cord decompression with the patient under general anesthesia. After explanation of the anesthesia plan, the patient verbalized understanding and consented to undergo awake fiberoptic intubation. Her American Society of Anesthesiologists Physical Status classification was I, her Mallampati classification was I, and she had a good mouth opening. On arrival in our operating room (3 hours from admission), noninvasive blood pressure (BP) monitoring with three-lead electrocardiography and pulse oximetry was established. The patient’s basal values were BP 120/60 mmHg, HR 75 beats per minute and peripheral capillary oxygen saturation of 100% while breathing room air. Intravenous access was secured with two 18-gauge intravenous cannulae, and the patient was given 10 ml/kg of isotonic saline solution. Cefazolin 2 g was also administered intravenously. After 5 minutes of preoxygenation with 100% oxygen and nebulization of lidocaine 2%, we carried out a superior laryngeal block, which was followed by nasal fiberoptic intubation. Tracheal intubation was easily performed with a 7.0-mm armored tube. Once the patient’s end-tidal carbon dioxide level confirmed placement, general anesthesia was induced with propofol 2.5 mg/kg, fentanyl 4 μg/kg, and rocuronium 0.6 mg/kg. Immediately after intubation, the patient’s BP was 130/70 mmHg, and her HR was 85 beat per minute. She was ventilated with a target tidal volume of 450 ml and a respiratory rate of 13 breaths per minute. Sevoflurane (1–1.5%) in a mixture of oxygen and air (1:2) was used for maintenance of anesthesia. A radial arterial line was placed for continuous monitoring of arterial BP and collection of arterial blood for blood gas analysis during surgery. We also inserted a urinary catheter.

**Fig. 2:**
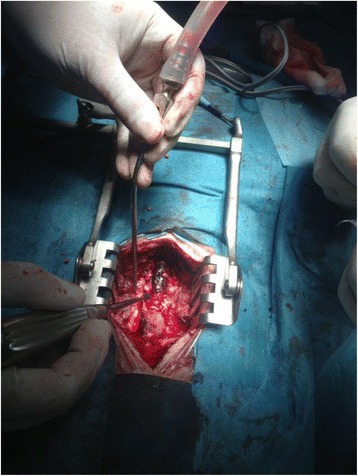
Operative finding: a hematoma on the dorsal aspect of the cervical spinal cord

The patient was turned to the prone position to allow a posterior cervical approach to the hematoma with appropriate padding and careful checking for pressure points. The endotracheal tube was rechecked for correct position by chest auscultation. The patient’s airway pressure was 22 cmH_2_O. Intraoperative fetal monitoring was not possible with the patient in this position. The patient’s mean BP was maintained between 85 and 95 mmHg. Perioperatively, blood gas analysis showed pH 7.4, partial pressure of carbon dioxide 36 mmHg, partial pressure of oxygen 135 mmHg, and bicarbonate 22.3 mmol/L. A laminectomy with evacuation of the epidural hematoma and decompression of the thecal sac was performed, followed by surgical hemostasis (Fig. 2). The operative findings did not reveal any arteriovenous malformation. The patient’s hemodynamic parameters remained stable during the procedure, and no vasopressors were required. Estimated blood loss was 350 ml, and the patient received a total of 1000 ml of isotonic crystalloid with adequate urine output.

At the end of the surgery, anesthetic gases were discontinued, and the patient was awakened. Immediately following surgery, a neurological examination of the patient was performed to evaluate her motor and sensory functions, which were identical to the preoperative findings. Postoperative analgesia consisted of intravenous paracetamol 1 g every 8 hours and intravenous nefopam 100 mg per 24 hours. Fetal ultrasound revealed a HR of 150 beats per minute. After discussion with the obstetrician, there was no indication for using tocolysis. The patient was continuously monitored for another 24 hours in the intensive care unit, and the postoperative period remained uneventful. She was transferred to the rehabilitation department where she underwent physical therapy. On postoperative day 2, she had slight sensation in both legs, and her muscle power showed a gradual improvement in the upper limbs (3/5 and 4/5, respectively, in the right and left upper limbs). After 1 month, power had improved to grade 4 in her left leg and to grade 3 in her right leg, and she was able to move her arms freely.

## Discussion

Spontaneous spinal epidural hematoma (SSEH) is a very uncommon medical condition in obstetrical patients [1, 2]. It is spontaneous in the absence of trauma, arteriovenous malformation, blood disorders, and epidural puncture [1, 3]. It has been suggested that raised intra-abdominal pressure forces venous return from the pelvis and abdomen to the vertebral venous plexus. Rupture of these veins within the negative pressure compartment of the epidural space explains the hemorrhage [1, 3, 4]. It has also been suggested that structural vessel changes induced by estrogen and progesterone excess [5], along with various anatomical factors [6–8], may favor SSEH occurrence.

SSEH is most often located in the thoracic region because of the rich epidural plexus present in this region. Only ten cases of pregnant women with SCEH have been described in the literature to date (Table 1). The SSEH is usually posterior or posterolateral to the thecal sac [9–12].

**Table 1:** Clinical characteristics, surgical management, and outcomes of published cases

Author [reference]	Age, years	GW	Time from ND to decompression	Symptoms	Level	Timing of decompression related to delivery	Surgical positioning	Outcome (recovery)
Mahieu *et al*. [1]	26	30	3 hours	Neck pain Brown-Séquard syndrome	C3–T1	After CS	?	Complete
Singh *et al*. [2]	25	31	30 hours	Interscapular pain Quadriplegia	C3–C7	Before VD	?	Complete
Yonekawa *et al*. [6]	20	37	14 hours	Neck pain Paraplegia	C4–C6	Before VD	Sitting position	No recovery
Wang *et al*. [13]	29	40	6 hours	Neck pain Quadriplegia	C5–C7	After CS	Prone position	Mild (impaired sensation in fingers)
Iwatsuki *et al*. [15]	27	37	Spontaneously resolved	Left shoulder pain	C4-T1	Abstention	Not done	Spontaneous recovery
Masski *et al*. [20]	27	41	12 hours	Neck and arm pain Quadriplegia	C7–T2	After CS	Prone position	No recovery
Binnert *et al*. [28]	28	37	120 hours	Interscapular pain Paraplegia	C7–T1	After VD	Sitting position	No recovery
Matsubara *et al*. [29]	36	16	9 hours	Interscapular pain Quadriplegia	C3–C7	Before CS	?	No recovery
Tada *et al*. [30]	26 21	31 39	30 hours 12 hours	Paraparesis Quadriparesis	C4–T2 C5–T2	After CS After CS	Prone position Prone position	Complete Complete
Our patient	35	21	28 hours	Neck and interscapular pain Quadriplegia	C3–C6	Before CS	Prone position	Mild

*Abbreviations: CS* Cesarean section, *GW* Gestational weeks, *ND* Neurological deficit, *VD* Vaginal delivery

Urgent surgical decompression and clot removal is the gold standard [13, 14], but some patients may be managed conservatively [15–18]. Incomplete preoperative sensorimotor loss and surgery within 36 hours of onset correlate with a favorable outcome [19].

The anesthetic management of SCEH in pregnancy is not well described. General anesthesia is necessary for cervical localizations, and the difficulty concerns airway management of these patients. Masski *et al*. reported a case of SCEH in a pregnant woman who was under general anesthesia using a rapid sequence technique with cricoid pressure [20]. The patient’s head was maintained in neutral position without a collar by a third person. Manual in-line stabilization (MILS) was used during direct laryngoscopy and intubation to minimize the risk of further cord compression. However, the MILS technique does not eliminate cervical spine movement [21], and it is known that MILS reduces mouth opening and leads to a poor laryngoscopic view [22].

In our patient, we used a different approach. We thought that conventional endotracheal intubation with direct laryngoscopy could have aggravated the injury and exposed the patient to a marked sympathetic response. Awake fiberoptic bronchoscopy was deemed most appropriate.

The management of acute spinal cord decompression should also include measures to preserve spinal cord perfusion [23] and adequate uterine blood flow [24] by ensuring volume expansion, using anesthetics that respect the hemodynamic status and administering vasopressors if needed (Table 2). Invasive BP monitoring with an indwelling arterial catheter is useful. Cardiac dysfunction secondary to loss of cardiac sympathetic tone is another reason for using invasive monitoring.

**Table 2:** Anesthetic management of published cases of spontaneous spinal epidural hematoma

Author [reference]	Localization	Induction of anesthesia	Intubation	Maintenance of anesthesia	Hemodynamic support	Outcome
Jo *et al*. [25]	T1–T5	Propofol 100 mg and rocuronium 50 mg	Direct laryngoscopy	Sevoflurane (1–1.5%) in 60% oxygen and air	Dopamine 5–10 μg/kg/minute (5 days)	Complete recovery
Doblar and Schumacher [4]	T6–T9	Etomidate 12 mg and succinylcholine 120 mg	Direct laryngoscopy	Isoflurane at 0.25–0.5 MAC in oxygen and nitrous oxide	Phenylephrine infusion (7 days)	Mild recovery
Masski *et al*. [20]	C7–T2	Thiopental 5 mg/kg and rocuronium 0.8 mg/kg	MILS + direct laryngoscopy	Not precise	Not used	No recovery
Our patient	C3–C6	nebulization of lidocaine 2% and superior laryngeal block propofol 2.5 mg/kg, fentanyl 4 μg/kg, and rocuronium 0.6 mg/kg	Awake fiberoptic intubation	Sevoflurane (1–1.5%) in 50% oxygen and air	Not used	Mild recovery

*MAC* Minimum alveolar concentration, *MILS* Manual in-line stabilization

Lateral positioning was considered for our patient, but the neurosurgeons expressed their view that this position would have made the surgery technically difficult with an increase of the surgery duration and bleeding. Prone position is limited by the difficulty in monitoring the fetal HR and uterine activity. According to the American Congress of Obstetricians and Gynecologists, the decision whether to use intraoperative fetal monitoring should be determined by a multidisciplinary team and based on each patient’s unique circumstances and the surgery to be performed [25]. After a multidisciplinary team discussion, the prone position was selected for our patient to allow optimal surgical access. Careful positioning for laminectomy was done with help and guidance of the neurosurgical team. For this position, an armored tracheal tube is necessary.

Finally, at the end of the surgery, airway edema is one of the major concerns in patients undergoing surgery in prone position [26, 27], combined with effects of the gravid state and parenteral fluid administration. The precaution of direct laryngoscopy prior to extubation is justified. During the postoperative period, patients need expensive health care. Postoperative documentation of motor and sensory function is essential for these patients, and they should be monitored for any hemodynamic alterations in a high-dependency unit.

## Conclusions

This case report highlights the management of an uncommon medical condition in obstetrical patients. The potential for difficult airway management with the patient under general anesthesia is one of the major concerns. Anesthetic management should also include measures to maintain the mean arterial pressure to improve spinal cord perfusion, as well as special considerations regarding positioning and postoperative management.

## Abbreviations

BP: Blood pressure
CS: Cesarean section
GW: Gestational weeks
HR: Heart rate
MAC: Minimum alveolar concentration
MILS: Manual in-line stabilization
ND: Neurological deficit
SCEH: Spontaneous cervical epidural hematoma
SSEH: Spontaneous spinal epidural hematoma
VD: Vaginal delivery

## References

[1] Mahieu X, Kridelka F, Pintiaux A, Hans P, Brichant JF. Spontaneous cervical extradural hematoma in a pregnant woman. J Gynecol Obstet Biol Reprod (Paris). 1994;23:99–102. 8157901

[2] Singh DP, Lamtha SC, Kumar S. Spontaneous spinal haematoma during pregnancy. J Assoc Physicians India. 2009;57:540–2. 20329420

[3] Loo CC, Dahlgren G, Irestedt L. Neurological complications in obstetric regional anaesthesia. Int J Obstet Anesth. 2000;9:99–124. 10.1054/ijoa.1999.0347 15321097

[4] Doblar DD, Schumacher SD. Spontaneous acute thoracic epidural hematoma causing paraplegia in a patient with severe preeclampsia in early labor. Int J Obstet Anesth. 2005;14:256–60. 10.1016/j.ijoa.2004.09.009 15935638

[5] Cywinski JB, Parker BM, Lozada LJ. Spontaneous spinal epidural hematoma in a pregnant patient. J Clin Anesth. 2004;16:371–5. 10.1016/j.jclinane.2003.08.007 15374559

[6] Yonekawa Y, Mehdorn HM, Nishikawa M. Spontaneous spinal epidural hematoma during pregnancy. Surg Neurol. 1975;3:327–8. 1162587

[7] Carroll SG, Malhotra R, Eustace D, Sharr M, Morcos S. Spontaneous spinal extradural hematoma during pregnancy. J Matern Fetal Med. 1997;6:218–9. 10.1002/(SICI)1520-6661(199707/08)6:4<218::AID-MFM6>3.0.CO;2-M 9260119

[8] Groen RJ, Ponssen H. The spontaneous spinal epidural hematoma: a study of the etiology. J Neurol Sci. 1990;98:121–38. 10.1016/0022-510x(90)90253-j 2243224

[9] Alexiadou-Rudolf C, Ernestus RI, Nanassis K, Lanfermann H, Klug N. Acute nontraumatic spinal epidural hematomas: an important differential diagnosis in spinal emergencies. Spine. 1998;23:1810–3. 10.1097/00007632-199808150-00018 9728384

[10] Fukui M, Swarnkar A, Williams R. Acute spontaneous spinal epidural hematomas. AJNR Am J Neuroradiol. 1999;20:1365–72. PMC7055980 10472999

[11] Joseph A, Vinen J. Acute spinal epidural hematoma. J Emerg Med. 1993;11:437–41. 10.1016/0736-4679(93)90247-5 8228107

[12] Groen R, van Alphen A. Operative treatment of spontaneous spinal epidural hematoma: a study of the factors determining postoperative outcome. Neurosurgery. 1996;39:494–502. 10.1097/00006123-199609000-00012 8875479

[13] Wang P, Xin XT, Lan H, Chen C, Liu B. Spontaneous cervical epidural hematoma during pregnancy: case report and literature review. Eur Spine J. 2011;20 Suppl 2:S176–9. 10.1007/s00586-010-1484-9 PMC3111511 20563824

[14] Szkup P, Stoneham G. Case report: spontaneous spinal epidural hematoma during pregnancy: case report and review of the literature. Br J Radiol. 2004;77:881–4. 10.1259/bjr/58217479 15483004

[15] Iwatsuki K, Deguchi M, Hirata H, Kanamono T. Spontaneously resolved recurrent cervical epidural hematoma in a 37-week primigravida. Global Spine J. 2015;5:e44–7. 10.1055/s-0034-1398489 PMC4577326 26430600

[16] Dam-Hieu P, Mihalescu M, Tadié M. Spontaneous regression of paraplegia caused by spontaneous cervico-thoracic epidural hematoma [in French]. Neurochirurgie. 2001;47:442–4. 11493875

[17] Ohayon L, Gorhan C, Soto-Ares G, Reyns N, Pruvo JP. Acute spinal epidural and subdural hematomas. J Radiol. 2003;84:50–3. 12637888

[18] Hentschel SJ, Woolfenden AR, Fairholm DJ. Resolution of spontaneous spinal epidural hematoma without surgery: report of two cases. Spine. 2001;26:525–7. 10.1097/00007632-200111150-00025 11707724

[19] Lawton MT, Porter RW, Heiserman JE, Jacobowitz R, Sonntag VK, Dickman CA. Surgical management of spinal epidural hematoma: relationship between surgical timing and neurological outcome. J Neurosurg. 1995;83:1–7. 10.3171/jns.1995.83.1.0001 7782824

[20] Masski G, Housni B, Ibahiouin K, Miguil M. Spontaneous cervical epidural hematoma during pregnancy. Int J Obstet Anesth. 2004;13:103–6. 10.1016/j.ijoa.2003.09.006 15321414

[21] Lennarson PJ, Smith DW, Sawin PD, Todd MM, Sato Y, Traynelis VC. Cervical spinal motion during intubation: efficacy of stabilization maneuvers in the setting of complete segmental instability. J Neurosurg. 2001;94(2 Suppl):265–70. 10.3171/spi.2001.94.2.0265 11302629

[22] Heath KJ. The effect of laryngoscopy of different cervical spine immobilisation techniques. Anaesthesia. 1994;49:843–5. 10.1111/j.1365-2044.1994.tb04254.x 7802175

[23] Vale FL, Burns J, Jackson AB, Hadley MN. Combined medical and surgical treatment after acute spinal cord injury: results of a prospective pilot study to assess the merits of aggressive medical resuscitation and blood pressure management. J Neurosurg. 1997;87:239–46. 10.3171/jns.1997.87.2.0239 9254087

[24] Braveman FR, Scavone BM, Wong CA, Santos AC. Obstetrical anesthesia. In: Barash PG, Cullen BF, Stoelting RK, Cahalan M, Stock MC, editors. Clinical anesthesia. 6th ed. Philadelphia: Wolters Kluwer/Lippincott Williams & Wilkins; 2009. p. 1137–67.

[25] Jo YY, Lee D, Chang YJ, Kwak HJ. Anesthetic management of a spontaneous spinal-epidural hematoma during pregnancy. Int J Obstet Anesth. 2012;21:185–8. 10.1016/j.ijoa.2011.12.008 22341893

[26] Sinha A, Agarwal A, Gaur A, Pandy CK. Oropharyngeal swelling and macroglossia after cervical spine surgery in the prone position. J Neurosurg Anesthesiol. 2001;13:237–9. 10.1097/00008506-200107000-00010 11426099

[27] McAllister RG. Macroglossia—a positional complication. Anesthesiology. 1974;40:199–200. 10.1097/00000542-197402000-00022 4812721

[28] Binnert D, Thierry A, Michiels R, Soichot P, Perrin M. Presentation of a new case of spontaneous spinal extradural hematoma observed during labor [in French]. J Med Lyon. 1971;52:1307–9. 5167760

[29] Matsubara S, Inoue H, Takamura K, Kimura A, Okuno S, Fujita A et al. Spontaneous spinal epidural hematoma at the 16th week of a twin pregnancy. J Obstet Gynaecol Res. 2011;37:1466–9. 10.1111/j.1447-0756.2010.01522.x 21564404

[30] Tada S, Yasue A, Nishizawa H, Sekiya T, Hirota Y, Udagawa Y. Spontaneous spinal epidural hematoma during pregnancy: three case reports. J Obstet Gynaecol Res. 2011;37:1734–8. 10.1111/j.1447-0756.2011.01592.x 21790886

